# Gender-related association among childhood maltreatment, brain structure and clinical features in bipolar disorder

**DOI:** 10.1016/j.euroneuro.2022.07.186

**Published:** 2022-08-28

**Authors:** Lejla Colic, Alexis Clark, Anjali Sankar, Durga J. Rathi, Danielle A. Goldman, Jihoon A. Kim, Luca M. Villa, E. Kale Edmiston, Elizabeth T.C. Lippard, Brian Pittman, R. Todd Constable, Carolyn M. Mazure, Hilary P. Blumberg

**Affiliations:** a Department of Psychiatry, Yale School of Medicine, USA; b Department of Psychiatry and Psychotherapy, Jena University Hospital, Germany; c German Center for Mental Health, Halle/Jena/Magdeburg, Germany; d Department of Neurology and Neurobiology Research Unit, Copenhagen University Hospital, Denmark; e Interdepartmental Neuroscience Program, Yale School of Medicine, USA; f Department of Psychiatry, University of Oxford, UK; g Department of Psychiatry, University of Pittsburgh, USA; h Department of Radiology and Biomedical Imaging, Yale School of Medicine, USA; i Department of Psychiatry and Behavioral Sciences and Institute of Early Life Adversity Research, Dell Medical School, University of Texas, USA; j Women’s Health Research at Yale, Yale School of Medicine, USA; k Child Study Center, Yale School of Medicine, USA

**Keywords:** Bipolar disorder, Child abuse, Child neglect, Gender differences, Hippocampus, Prefrontal cortex

## Abstract

Bipolar disorder (BD) and exposure to childhood maltreatment (CM), which is present at high rates in BD, are both associated with hippocampus and prefrontal cortex structural alterations thought to contribute to clinical features. Gender-related differences are implicated in BD for CM exposure, brain structure and clinical features. However, relationships among these factors in BD are understudied. This study aimed to investigate associations among gender, CM, hippocampus and prefrontal gray matter structure and clinical features in BD. Childhood trauma questionnaire, structured clinical assessments and 3 Tesla structural magnetic resonance imaging were obtained for 236 adults (18-63 years, 32.0 ± 12.6): 119 with BD (58.8% women) and 117 healthy controls (HCs, 50.4% women). Women with BD reported higher CM severity than men with BD and HCs (*B*=−14.34, 95% confidence intervals (CI)[−22.71,−5.97], *p*<.001). CM and gender showed a significant interaction for left hippocampus (*B*=−7.41, 95% CI[−14.10,−0.71], *p*<.05); CM severity was negatively associated with left hippocampus only in women with BD. In women with BD, CM was associated with post-traumatic stress disorder comorbidity (*B* = 25.68, 95% CI[15.11,36.25], *p*<.001). In men with BD, CM severity was associated with lower left frontal pole (*B*=−0.71, 95% CI[−1.14,−0.28], *p*<.05) and right superior frontal (*B*=−17.78, 95% CI[−30.66,−4.90], *p*<.05) surface area; the latter related to earlier age of first mood symptoms (*B* = 33.97, 95% CI[7.61, 60.33], *p*<.05). Findings support gender-related effects of CM on frontotemporal structure and clinical features of BD. The findings bring novel perspectives for gendered pathophysiological models of effects of CM in BD.

## 1. Introduction

Childhood maltreatment (CM), which can comprise physical and emotional abuse and neglect and sexual abuse (Teicher et al., 2016), is an important risk factor for symptoms of bipolar disorder (BD) (Lippard and Nemeroff, 2020) and women with BD report higher rates of CM than men with BD (Etain et al., 2013; Monteleone et al., 2020). CM severity is associated with clinical features of BD, such as number of episodes, rates of comorbid post-traumatic stress disorder (PTSD) and suicide attempts (Agnew-Blais and Danese, 2016). Importantly, although BD prevalence rates are similar for women and men, the genders differ in clinical features (Diflorio and Jones, 2010). For example, women with BD have higher rates of comorbid PTSD (Baldassano et al., 2005) and suicide attempts (Tondo et al., 2016). Overlap between these clinical characteristics and the ones associated with CM, and higher rates of CM in women with BD, suggest that CM exposure may contribute to differences in clinical features between genders (Aas et al., 2016). However, the gender-related neurobiological mechanisms underlying CM’s effects on clinical features in BD have received little study, though their identification could contribute to much-needed improvements in treatment strategies.

Structural magnetic resonance imaging (MRI) studies of individuals who experienced CM both with and without BD have shown alterations in hippocampus and prefrontal cortex (PFC) (Edmiston et al., 2011; Moreno-López et al., 2020; Paquola et al., 2016). Evidence suggests associations between CM and the hippocampus and dorsal PFC (Teicher et al., 2006) that differ between genders (Edmiston et al., 2011; Pechtel and Pizzagalli, 2011). In BD, while amygdala and ventral PFC structural abnormalities have been observed consistently, hippocampus and dorsal PFC structural findings have varied (Blond et al., 2012; Strakowski et al., 2012) and the observed heterogeneity may be contributed to by gender (Jogia et al., 2012) and CM (Zovetti et al., 2022). Previous studies that focused on PFC volume may have been limited in detecting gender-related findings. Separately studying cortical surface area and thickness, which each contribute to volume measures but have different genetic and developmental associations (Winkler et al., 2010), may provide additional sensitivity. Surface area is especially implicated as it shows more variability during childhood, and may therefore be more susceptible to CM (Lyall et al., 2015). This is supported by a study of individuals with major depressive disorder that showed associations between higher CM severity and lower cortical surface area (Opel et al., 2019). Interestingly, hippocampus and dorsal PFC volume decreases are also thought to contribute to clinical features of BD (Frey et al., 2007), such as suicide attempts (Fan et al., 2019) and number of manic episodes (Lyoo et al., 2004).

Together, previous research raises the possibility that CM may alter hippocampus and dorsal PFC structure in individuals with BD in a gender-related manner and that these brain alterations are associated with clinical features. This study therefore aimed to examine the relationships among CM severity, gender, hippocampus and PFC structure, and clinical features in BD. We hypothesized that women with BD would report higher levels of CM compared to men with BD and a healthy control (HC) group. We further hypothesized that the CM severity would be associated with more severe clinical features. We expected that higher CM levels would be associated with lower hippocampus volume in women with BD and dorsal PFC surface area across genders. In an exploratory manner, we also investigated the association between the CM-related brain structure alterations and clinical features in each gender.

## 2. Experimental procedures

## 2.1. Participants

The study included 236 participants: 119 with BD and 117 HCs. The BD group comprised 70 women (58.8% of BDs, ages 18-58 years, mean age± standard deviation=30.1 ± 12.0) and 49 men (41.2%, ages 18-62 years, mean=31.9 ± 13.1); the HC group comprised 59 women (50.4%, ages 18-57 years, mean=31.7 ± 13.1) and 58 men (49.6%, ages 18-55 years, mean=34.7 ± 12.5). Inclusion criteria for all participants were ages between 18 and 60 years. Inclusion criteria for the BD group were meeting diagnostic criteria for BD according to the Diagnostic and Statistical Manual of Mental Disorders (Fourth ed. Text Revision; DSM-IV-TR), while for the HC group inclusion criteria were absence of any Axis I psychiatric disorders or first-degree relative with a major mood or psychotic disorder, assessed with the Family History Screen for Epidemiological Studies (Lish et al., 1995). Exclusion criteria for all participants included MRI contraindications and medical and neurological illnesses and conditions (including loss of consciousness >5 min) that could alter cerebral tissue, except treated thyroid disorders in 5 BD participants. On the scanning day, urine screening was negative in all participants for cannabis, cocaine, amphetamine, methamphetamine, methadone, opiates, phencyclidine, barbiturates, and benzodiazepines, and in female participants for pregnancy. Participants were recruited from the Yale University-affiliated clinical programs and the Greater New Haven, Connecticut community. All participants provided written informed consent in accordance with the Yale School of Medicine Human Investigation Committee institutional review board.

### 2.2. Clinical assessments

The presence or absence of psychiatric diagnoses, mood state and other clinical features (age of first mood symptoms, numbers of hospitalizations and episodes, history of rapid cycling and psychosis) were confirmed by the Structured Clinical Interview for DSM-IV Axis I disorder (SCID-IV) (First et al., 1994) and consensus diagnosis with licensed clinicians. For BD participants, current mood symptom severity was assessed with the Hamilton Depression Rating Scale 29-item version (HDRS) (Williams, 1988) (internal consistency, Cronbach’s alpha (*α*_C_ )=0.9) and Young Mania Rating Scale (YMRS) (*α*_C_=0.8) (Young et al., 1978). History of suicide attempts were defined as “actual” attempts using the Columbia Suicide History Form (Oquendo et al., 2003). Table 1 provides further details about participants’ clinical characteristics and Supplementary Table 1 provides detailed list of current medication. BD participants with a history of alcohol or substance abuse or dependence were included since use disorders are frequent comorbidities with BD (Swann, 2010), and were previously associated with CM in BD (Agnew-Blais and Danese, 2016). Participants were without history of alcohol or substance abuse within 3 months, or dependence within 6 months of study except 9 BD participants (2 females) with more recent alcohol and/or substance abuse or dependence (three <6 months, five <3 months, 1 current alcohol abuse with last use one week ago). For the 23 women and 5 men who met criteria for the PTSD, the reported stressor for PTSD occurred before age of 12 years for 15 (71.4%) women and 2 (40.0%) men.

### 2.3. Childhood maltreatment

Participants completed the Childhood Trauma Questionnaire (CTQ) (Bernstein et al., 2003), a 28-item, self-report questionnaire about childhood experience, with a total score (CTQ-tot; *α*_C_=0.8) that consists of five subtypes of CM each corresponding to a subscale: physical abuse (*α*_C_=0.9), emotional abuse (*α*_C_=0.9), sexual abuse (*α*_C_ >0.9), physical neglect (*α*_C_=0.7) and emotional neglect (*α*_C_=0.9). The threshold for severe CM is CTQ-tot≥69. Primary analyses of associations with clinical and neuroimaging features were done with the CTQ-tot scores, while the exploratory analyses were done with the subscale scores.

### 2.4. Structural magnetic resonance imaging acquisition and processing

#### 2.4.1. Acquisition

A high resolution, three-dimensional Magnetization Prepared Rapid Acquisition Gradient Echo (MPRAGE) T1-weighted sequence was used to acquire sagittal images on a 3-Tesla Siemens Trio MR scanner (Siemens, Erlangen, Germany) with parameters: repetition time=1500 ms, echo time=2.77 ms, matrix=256 × 256, field of view=256 × 256mm^2^ , 160 1 mm slices without gap for a voxel-size of 1mm^3^, and two averages.

#### 2.4.2. Processing

MRI data was processed following “Enhancing neuroimaging genetics through meta-analysis” (ENIGMA) consortium pipelines (http://enigma.usc.edu/). Segmentation was performed using the tool recon-all within the software FreeSurfer V.6 on a Linux (Centos6.9_x86_64) machine (Fischl, 2012).

#### 2.4.3. Brain region definition

Left and right hippocampus volumes were parcellated and extracted based on the Aseg Atlas (Fischl et al., 2002), and cortical regions based on the Desikan-Killiany Atlas (Desikan et al., 2006). Surface area and cortical thickness were assessed for left and right PFC regions: superior frontal, rostral middle frontal, frontal pole, rostral anterior cingulate and medial orbitofrontal. Prior to inclusion in the study analysis, segmentation accuracy was confirmed for all participants by visual inspection based on Enigma protocols.

### 2.5. Statistical analyses

#### 2.5.1. CM and clinical feature analysis

Continuous variables were examined for normality using Shapiro-Wilk normality test, histograms, and normal-probability plots. Comparisons between diagnostic and gender groups and between clinical variables within BD were performed using Wilcoxon rank sum and Fisher’s exact tests, as appropriate. CTQ-tot and subscale scores were compared with regression analysis, testing interaction between diagnostic and gender groups.

Associations between the CTQ-tot and clinical features were tested using linear regression. Models included gender, clinical feature, and their interaction. If the gender by clinical feature interaction was not significant it was dropped for parsimony. These analyses were performed in the overall BD sample for mood state (euthymic, depressed, manic/hypomanic, mixed), current symptom severity (HDRS and YMRS), age of first mood symptoms, and numbers of hospitalizations, depressive episodes and manic episodes, as well as any additional feature with >10 participants in each gender present or absent over the lifetime, i.e. rapid cycling, psychosis, suicide attempt, attention deficit hyperactivity disorder (ADHD), anxiety disorders other than PTSD, substance abuse and substance dependence. For features present in >10 participants in only one gender, main effects of clinical features were assessed in that gender. These included among women, PTSD, eating disorders and alcohol abuse, and among men, alcohol dependence. Main effects of gender and diagnosis, or interactions between them, for CTQ-tot were considered significant at a two-sided alpha threshold of 0.05 with a false discovery rate (FDR) correction for multiple comparisons (p_FDR_).

In women with BD, features associated with having CTQ-tot in the severe range (≥69; *n* = 17, 24.3%), compared to those associated with scores in the none-moderate range (25-68; *n* = 53, 75.7%), were explored using Wilcoxon rank sum and Fisher’s exact tests. This analysis was not performed for men as only 4 men were above the severe threshold.

#### 2.5.2. CM and imaging analysis

Within each diagnostic group three imaging measures served as dependent variables, hippocampus volumes, and PFC surface area and cortical thickness. Interaction effects between CTQ-tot and gender were tested, covarying for age and intracranial volume (for volume and surface area analyses). PFC surface area and thickness were first assessed using an omnibus multivariate analysis of covariance (MAN-COVA) that was followed by univariate analyses of covariance (ANCOVAs) to further assess regional and laterality effects. ANCOVAs were considered significant using a two-sided alpha threshold of 0.05 with FDR correction.

Additionally, imaging analyses were repeated after excluding the 9 participants with more recent alcohol/substance abuse/dependence. For regions that showed significant interactions, similar models among all participants were used to explore each of the 5 CTQ subscale scores, separately, within genders.

#### 2.5.3. Brain structure and clinical features analysis

In brain regions showing significant associations with the CTQ-tot, exploratory analyses tested relationships between structural measures in the regions and clinical features that also showed associations to CTQ-tot, i.e. mood state, HDRS and YMRS scores, age of first mood symptoms, numbers of hospitalizations, depressive episodes and manic episodes, and history of suicide attempts, anxiety disorders other than PTSD, substance abuse and substance dependence, and in women history of PTSD. Main effects of clinical features on hippocampus volume and PFC surface area controlling for CTQ-tot were explored, as well as an interaction term between CTQ-tot and clinical features. Age and intracranial volume (ICV) were covariates. These exploratory analyses were considered significant at an uncorrected two-sided alpha threshold of 0.05.

All analyses and graphics were conducted and generated using RStudio (V 1.1.463) and package ggplot2.

## 3. Results

### 3.1. Childhood maltreatment

BD women (*n* = 70) had significantly higher CTQ-tot scores than BD men (*n* = 49) and HC women (*n* = 58) and men (*n* = 59); interaction of diagnosis by gender: *B*=−14.34, standard error (SE)=4.25, 95% CI[−22.71,−5.97], *p*<.001; details for all subscales in Supplementary Table 2 ). The percentage of participants with severe CM was higher for BD women (*n* = 17, 24.3%) than BD men (*n* = 4, 8.2%) (*p*=.03, odds ratio (OR)=3.57, 95% CI[1.06,15.66]); no HC participant had severe CM. The BD and HC groups did not differ in gender composition (*p*=.24, OR=0.71, 95% CI[0.41,1.23]) or in age (p_Wilcoxon_=0.21, *W* = 6301, 95% CI[−4.27,0.65]). Genders also did not differ in age (p_Wilcoxon_=0.21, *W* = 7558.5, 95% CI[−0.78,4.52]).

### 3.2. Clinical features and childhood maltreatment

BD women had higher HDRS (p_Wilcoxon_=0.045, *W* = 1344.5, 95% CI[−6.00,−8.85^10^−6^]) and YMRS (p_Wilcoxon_=0.033, *W* = 1324.5, 95% CI[−4.00,−3.85^10^−6^]) scores, higher comorbid PTSD (*p*=.004, OR=4.26, 95% CI[1.42,15.61]), and higher current use of antidepressants (*p*=.017, OR=3.25, 95% CI[1.14,10.75]), compared to BD men (Table 1 and Supplementary Table 1).

Associations between clinical features and CTQ-tot scores are shown in Table 2. Significant findings within the BD group included higher CTQ-tot scores in participants in depressed or mixed states than euthymic participants, and in association with higher HDRS and YMRS scores. Lifetime clinical features significantly associated with higher CTQ-tot were earlier age of first mood symptoms, higher numbers of hospitalizations and of depressive episodes, and history of suicide attempts. Associations with elevated mood state, number of manic episodes, and substance abuse and substance dependence comorbidity approached significance (0.05 < p_FDR_ <0.1). No significant interactions between clinical features and gender were observed for CTQ-tot (all *p*>.05). In women with BD, PTSD was positively associated with CTQ-tot.

### 3.3. Clinical profile of women with severe CM

Compared to women with CTQ-tot scores <69 (hereafter ≤moderate), women with CTQ-tot score ≥69 (hereafter severe) had higher YMRS scores (≤moderate, mean=5.6 ± 5.6; severe, mean=9.3 ± 7.4; p_Wilcoxon_=0.05, *W* = 308, 95% CI[−7.00,7.68^10^−5^]). Approaching significance, women in the CM severe group had earlier age of first mood symptoms (≤moderate, mean=15.1 ± 7.6; severe, mean=11.7 ± 7.5; p_Wilcoxon_=0.054, *W* = 591.5, 95% CI[−7.81^10^−5^,7.00]), higher numbers of hospitalization (≤moderate, mean=2.1 ± 2.7; severe, mean=4.0 ± 4.8; p_Wilcoxon_=0.066, *W* = 318.5, 95% CI[−2.00,5.06^10^−5^]), and depressive episodes (≤moderate, mean=12.2 ± 8.1; severe, mean=16.2 ± 6.5; p_Wilcoxon_=0.073, *W* = 330.5, 95% CI[−11.00,5.38^10^−5^]). The BD women with severe CM also had a higher percentage of participants with lifetime comorbid PTSD (≤moderate, *n* = 10, 18.9%; severe *n* = 13, 76.5%; *p*<.001, OR=13.27, 95% CI[3.26,68.25]) and approaching significance higher percentage of participants with lifetime comorbid substance dependence (≤moderate, *n* = 6, 11.3%; severe *n* = 6, 35.3%; *p*=.058, OR=4.16, 95% CI[0.92,19.14]).

### 3.4. MRI findings

**Fig. 1:**
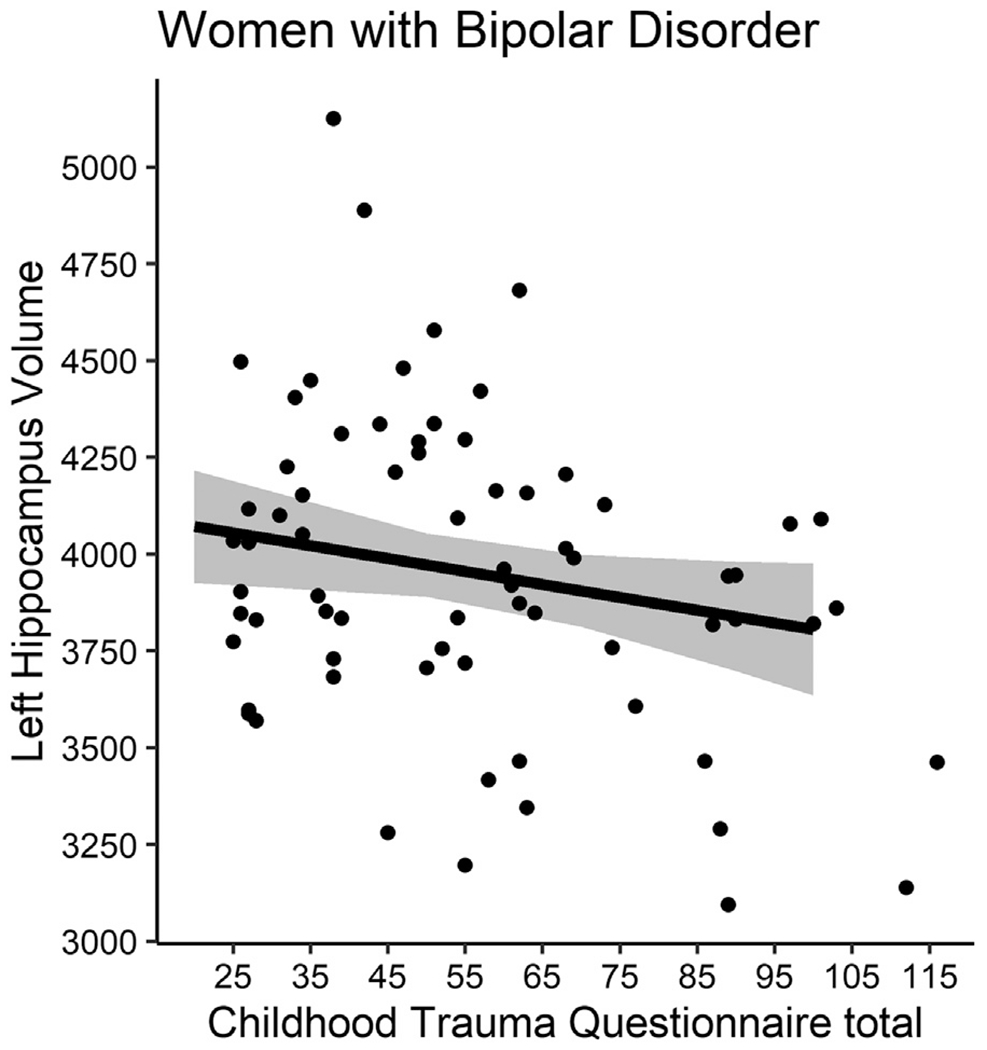
Association between total childhood trauma questionnaire scores and left hippocampus volume in women with bipolar disorder. A significant interaction between gender and total Childhood Trauma Questionnaire (CTQ-tot) scores was observed for left hippocampus volume (*B*=−7.41, SE=3.38, 95% CI[−14.10,−0.71], *p*=0.03). Women with bipolar disorder (*n* = 70) showed a negative association approaching significance between CTQ-tot scores and volume of the left hippocampus (*B*=−3.32, SE=1.71, 95% CI[−6.73,1.00], *p*=0.06). Regression line and confidence bands are adjusted for age and intracranial volume.

**Fig. 2:**
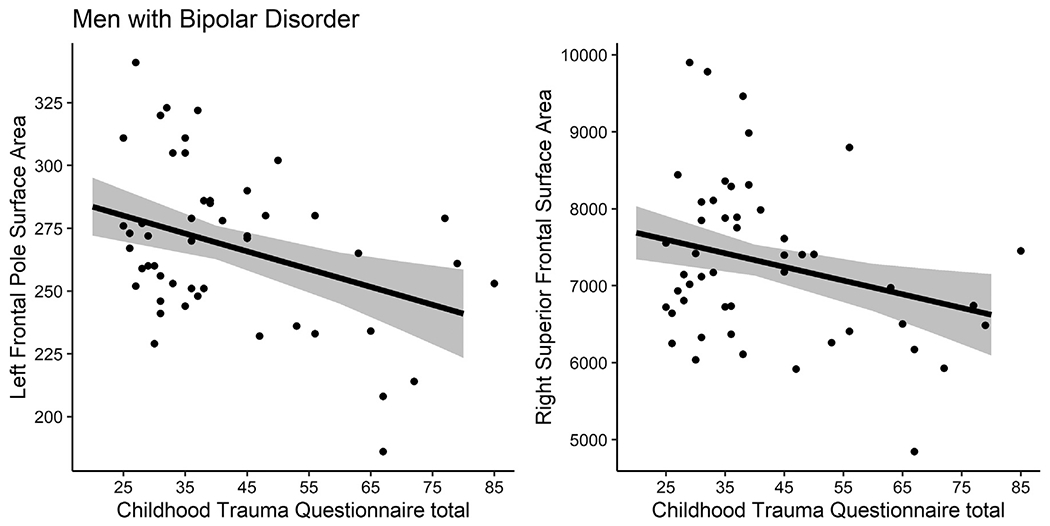
Associations between total childhood trauma questionnaire scores and left frontal pole and right superior frontal surface area in men with bipolar disorder. An interaction between gender and total Childhood Trauma Questionnaire (CTQ-tot) scores was observed for left frontal pole surface are (*B* = 0.64, SE=0.27, 95% CI[0.11, 1.17], p_uncorr_=0.020) and right superior frontal cortex surface area (*B* = 18.79, SE=7.11, 95% CI[4.71,32.87], p_uncorr_=0.009). Men with bipolar disorder (*n* = 49) showed a significant negative association between CTQ-tot scores and surface area of the left frontal pole (*B*=−0.71, SE=0.21, 95% CI[−1.14,−0.28], *p*=0.002) and right superior frontal cortex (*B*=−17.78, SE=6.39, 95% CI[−30.66,−4.90], *p*=0.008). Regression line and confidence bands are adjusted for age and intracranial volume.

A significant interaction between gender and CTQ-tot was observed for left hippocampus volume (*B*=−7.41, SE=3.38, 95% CI[−14.10,−0.71], *p*=.03). BD women showed a negative association between the two measures (*n* = 70, *B*=−3.32, SE=1.71, 95% CI[−6.73,1.00], *p*=.06; Fig. 1). The direction was also negative for right hippocampus; however, interaction was not observed at a significant level (*B*=−5.69, SE=3.61, 95% CI[−12.84,1.45], *p*=.12). Interaction of CTQ-tot and gender for PFC surface area in the BD group was below significance in the MANCOVA model (Pillai test=0.14, F_10,104_=1.71, *p*=.088). Follow-up ANCOVAs revealed significant effects at the uncorrected level for the left frontal pole (*B* = 0.64, SE=0.27, 95% CI[0.11, 1.17], p_uncorr_=0.020, p_FDR_=0.099) and the right superior frontal (*B* = 18.79, SE=7.11, 95% CI[4.71,32.87], p_uncorr_=0.009, p_FDR_=0.093) regions. For PFC interactions, a significant negative slope was observed in BD men (*n* = 49; left frontal pole: *B*=−0.71, SE=0.21, 95% CI[−1.14,−0.28], *p*=.002; right superior frontal gyrus: *B*=−17.78, SE=6.39, 95% CI[−30.66,−4.90], *p*=.008); Fig. 2). After exclusion of the 9 participants with more recent alcohol/substance abuse/dependence, the main neuroimaging results were similar (see Supplementary material).

Omnibus models testing of cortical thickness in the BD group, and all three imaging measures in the HC group, had no statistically significant findings (ps’>0.16).

For the exploratory CTQ subscale analyses in BD participants, brain regions and subscales that showed significant associations were: for women left hippocampus with sexual abuse; for men left frontal pole with emotional neglect, physical neglect and emotional abuse, and right superior frontal surface area with emotional neglect, physical abuse and sexual abuse, for statistical details see Supplementary material.

### 3.5. Association between the brain structure and clinical features

**Fig. 3:**
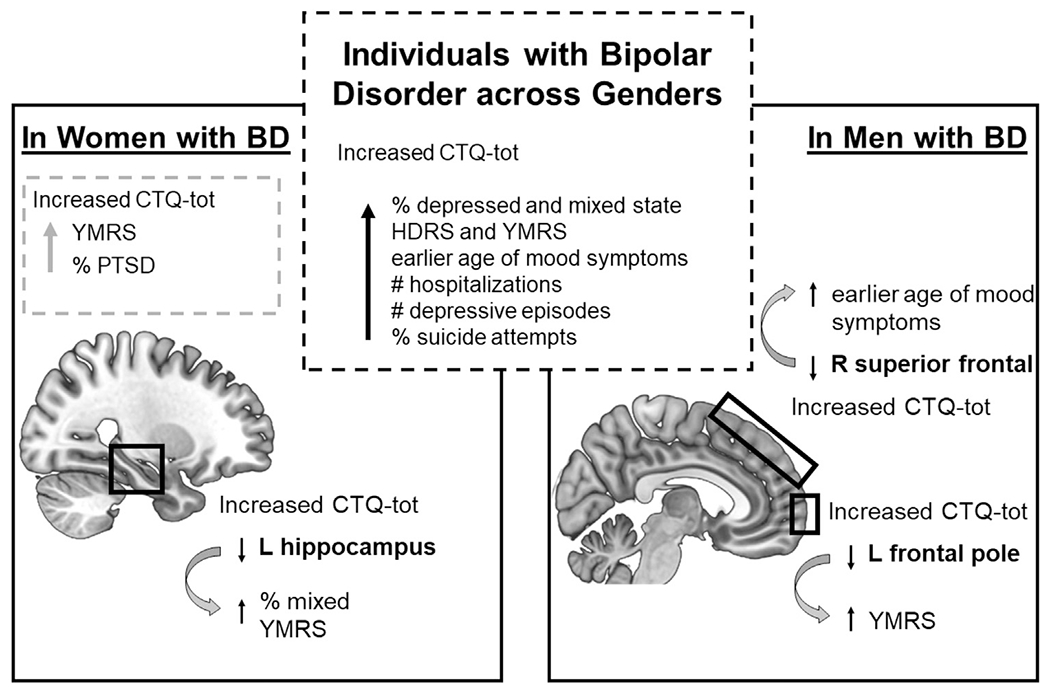
Schematic summary of the effects of childhood maltreatment by gender. Across genders, higher CTQ-tot is associated with clinical features; in women with BD, CTQ-tot is associated with PTSD, YMRS and lower left hippocampal volume, which also relates to mixed state and YMRS; in men with BD, higher CTQ-tot was associated with lower left frontal pole surface area, also associated with YMRS, and right superior frontal surface area, associated with earlier age of first mood symptoms. Abbreviations: CTQ-tot= total score of Childhood Trauma Questionnaire; HDRS= total score of 29-item Hamilton depression rating scale; *L*= left; PTSD= post-traumatic stress disorder; *R*= right; YMRS = total score of Young mania rating scale.

In women with BD, left hippocampus volume was lower if they were in mixed states than in a euthymic state and in association with higher YMRS scores. For BD men, left frontal pole surface area was lower in association with higher YMRS scores; right superior frontal surface area was lower in association with earlier age of first mood symptoms (ps’<0.05). Detailed results are summarized in Supplementary Table 3. Interaction models between clinical features and CTQ-tot were not significant (ps’>0.1). Fig. 3 summarizes all results.

## 4. Discussion

This paper aimed to investigate relationships among gender, CM, brain structure and clinical features of BD. CM severity was significantly higher in women with BD than in males with BD and HC women and men. The CM findings in women with BD are consistent with a recent report of higher levels of CTQ-tot in women with BD when compared to men with BD, especially for the sexual abuse subscale (Monteleone et al., 2020). Taken together with our findings, this suggests that women with BD might be exposed to more CM. It is also possible that women with BD more readily disclose such experiences; however, PTSD and other trauma research suggest that the gender differences are not due to reporting bias (Chung and Breslau, 2008). For the BD participants across genders, this study was consistent with previous reports of clinical features being associated with higher self-reported CM exposure, including increased numbers of hospitalizations and depressive episodes, younger age of first mood symptoms, and history of suicide attempts (Agnew-Blais and Danese, 2016). Higher CTQ-tot scores were furthermore associated with current depressive and mixed states and higher HDRS and YMRS scores. Negative mood states may increase reporting negative autobiographical events (Dalgleish and Werner-Seidler, 2014). However, a prospective study found that individuals with BD and CM tend to have higher symptom severity more frequently (Leverich et al., 2002). In women with BD, CM severity was also related to PTSD comorbidity. The association among severity of CM and clinical features suggest that individuals with BD maltreated in youth may be at higher risk for certain clinical features, and a recent review highlighted the need for including evaluation of CM to improve mental health care (Teicher et al., 2022). Further, this study also revealed findings that were significant only in women or only in men (Fig. 3), supporting the need to also include considerations of gender in clinical evaluations of BD.

### 4.1. CM, brain structure and clinical features in women with BD

Consistent with previous studies reporting gender differences in associations among severe CM, stress-related HPA axis changes and hippocampus structural alterations (McEwen and Gianaros, 2010), in women with BD, higher CTQ-tot scores were negatively associated with left hippocampus volume. It is possible that CM leads to stronger effects on hippocampus structure in females with BD, such as by interacting with the neurodevelopmental pathophysiology of BD. Lower hippocampus volumes were found in girls but not boys ages 6-17 years with BD (Frazier et al., 2008), suggesting early structural vulnerability in the hippocampus in females with BD that may be further exacerbated by CM. As BD females had the highest CM levels in this study, it is also possible that there is a CM threshold over which the hippocampus is more likely to show alterations. We previously reported that in adolescent girls under age 18 years, who did not meet DSM-IV criteria for a psychiatric disorder, higher CTQ-tot was associated with lower hippocampus volume (Edmiston et al., 2011). In the older HC participants of the present study, this association was not significant. This suggests CM-related changes in the hippocampus in girls may be present before full brain maturation and predispose to risk for affective difficulties which may fully emerge after young adulthood, the epoch of highest risk for BD onset. From among the five CTQ subscales, sexual abuse severity was the one that was significant in its negative association with left hippocampus volume in BD women. Previous reports in women without psychiatric disorders or with major depressive disorder also showed lower hippocampus volume was particularly associated with childhood sexual abuse (Andersen et al., 2008; Dannlowski et al., 2012; Vythilingam et al., 2002). This suggests that exposure to sexual abuse in childhood may have particularly robust effects in altering hippocampus structure, especially for women with BD. The left hippocampus volume was also related to the presence of mixed episode and higher current mania while controlling for CTQ-tot scores. We speculate that the CM-related changes in hippocampus may confer vulnerability for higher elevated mood symptom severity.

### 4.2. CM, brain structure and clinical features in men with BD

In men with BD, higher CTQ-tot scores were associated with lower surface area in the left frontal pole. Lower frontal pole surface area was also significantly associated with higher emotional neglect and abuse and physical neglect subscale scores, as well as mania severity. Based on these findings and the literature, we speculate that emotional subtypes of CM may contribute to changes in frontal pole structure that may impair mood and emotion regulation in BD (Altshuler et al., 2008; Bludau et al., 2014) Effects of mood state on brain structure were reported previously for frontal regions, including frontal pole (Brooks Iii et al., 2009), and they were suggested to be a result of mood differences in metabolism or medication, although effects of gender and CM were not assessed. CTQ-tot scores were additionally associated with lower right superior frontal surface area, which was associated with emotional neglect, and physical and sexual abuse, subscale scores. Previous studies reported changes in volume for this frontal area in adults who reported CM (Chaney et al., 2014) and physically abused children (Hanson et al., 2010), but these studies did not investigate the relationship of gender. Earlier age of first mood symptoms was also related to lower right superior frontal surface area. It was suggested that early and late onset of BD have distinct neuropathological features (Bolton et al., 2021) and are related to differences in frontal cortical thickness (Oertel-Knochel et al., 2015). We speculate that there may be relationships between CM exposure and the pathophysiology underlying different ages of onset, but large studies with balanced age of onset groups are warranted.

A possible explanation for the PFC effects in the males not seen in the females is that PFC surface area expansion peaks later in males (Koolschijn and Crone, 2013), thus there could be an extended period in childhood when CM can affect PFC surface area in males. The findings in this study were in dorsal and rostral PFC areas, which can be the PFC areas latest to mature (Gogtay et al., 2004). In a large brain structure study of BD, lower cortical thickness in BD appeared to relate to the disorder (Hibar et al., 2018), whereas a study of CM showed associations between lower cortical surface areas and CTQ-tot scores (Opel et al., 2019), suggesting that cortical surface area may be especially sensitive to environmental exposure such as CM.

### 4.3. Limitations and suggestions for future research

The sample size was powered to detect medium effect sizes for this study’s research questions. Large longitudinal studies are needed to detect small effect sizes, and to dissect prospective relationships between CM, brain structure, and occurrences and changes in clinical features in a controlled manner. The CTQ scores relied on self-reporting and did not allow assessment of the age when the CM occurred. We did not observe significant associations between CTQ-tot and the brain measures in HCs. Both HC men and women had minimal CTQ scores, similar to findings from general population (Gerdner and Allgulander, 2009), and thus ability to detect associations with brain structure in HCs may have been limited by low variance. Future studies may consider enriching HC samples for higher levels of CM. Ability to detect associations with CM in men with BD, relative to women with BD, may also have been diminished by the lower levels of CM reported by the men. We did not evaluate current levels of stress. It is known that CM disrupts brain and bodily systems that are involved in response and regulation of stress (McEwen, 2000), especially in social situations (Fares-Otero and Martinez-Aran, 2022), and future studies should evaluate both CM and proximal stressors to elucidate each of their effects on brain and clinical features of BD. Exploration of potential effects of medication was limited, as BD participants are often on more than one current medication that was not systematically controlled for (e.g., active dosage and blood levels) and reporting on lifetime medication may be biased.

In summary, the results support hypotheses that women with BD report high levels of CM severity, that the severity of CM is associated with adverse clinical features across genders, and with structural alterations in the hippocampus in women and in PFC surface area in men with BD, which relate to mood severity (hippocampus in women and frontal pole surface area in men) and age of onset of mood symptoms (superior frontal surface area in men). The findings support the notion that the CM represents an important neuropathophysiological factor to consider in clinical care alongside gender. Future research focused on investigating gender as a moderating factor in the study of effects of CM on neuropathophysiological and clinical features of BD is warranted.

## 5. Contributors

LC made substantial contributions to the analysis of data, interpretation of findings, drafting of the manuscript and revising it critically for intellectual content. AC substantially contributed to the analysis of data, interpretation of findings and critical revisions of the manuscript. AS, EKE, ETCL, DG, LMV, DJR, JAK substantially contributed to the interpretation of findings, and critical revisions of the manuscript. BP provided statistical expertise on the analyses and contributed to the interpretation of findings and critical revisions of the manuscript. RTC made substantial contributions to the design of the study, interpretation of findings and critical revisions of the manuscript. CMM made substantial contributions to the design of the study, interpretation of findings and critical revisions of the manuscript. HPB made substantial contributions to the conception and design of the study, analysis of data, interpretation of findings and critical revisions of the manuscript.

## Supplementary Material

SI Supplementary Table 1 Medication of the Participants with Bipolar Disorder.
Supplementary Table 2 Total score and subscale scores of Childhood Trauma Questionnaire; descriptive and statistical comparison for diagnosis and gender.
Supplementary Table 3 Significant results of the association between the clinical features and brain regions by gender.

## Figures and Tables

**Table 1:** Clinical characteristics of the participants with bipolar disorder.

Clinical features	Women (*n* = 70)	Men (*n* = 49)	Statistical comparison
*Mood Sate [n (%)]*			
Euthymic /	26 (37.2%) /	27 (55.1%) /	*p*= .24, *χ*^2^(3)= 4.27
Depressed /	22 (31.4%) /	11 (22.4%) /	
Elevated /	11 (15.7%) /	7 (14.3%) /	
Mixed	11 (15.7%)	4 (8.2%)	
*Mood symptom severity [mean ± SD]*			
HDRS	11.9 ± 10.4	8.0 ± 8.7	***p*= .045, *W* = 1344.5, 95% CI [−6.00, −8.85^10^−6^]** *
YMRS	6.5 ± 6.2	4.1 ± 4.8	***p*= .033, *W* = 1324.5, 95% CI [−4.00, −3.85^10^−6^]** *
*Clinical features [mean ± SD]*			
Age of first mood symptoms	14.3 ± 7.6	16.7 ± 9.9	*p*= .19, *W* = 1956.00, 95% CI [−1.00, 4.00]
# hospitalizations	2.6 ± 3.4	2.1 ± 2.4	*p*= .51, *W* = 1594.50, 95% CI [−0.19, 1.40^10^−5^]
# depressive episodes	13.2 ± 7.9	11.4 ± 8.4	*p*= .20, *W* = 1492.50, 95% CI [−2.00, 2.32^10^−5^]
# manic episodes	9.7 ± 9.2	10.8 ± 8.3	*p*= .25, *W* = 1920.00, 95% CI [−5.55^10^−5^, 3.00]
*Clinical features [n (%)]*			
Rapid cycling	26 (37.1%)	15 (30.6%)	*p*= .56, OR= 1.34, 95% CI [0.58, 3.16]
Lifetime psychosis	25 (35.7%)	16 (32.6%)	*p*= .85, OR= 1.14, 95% CI [0.50, 2.68]
Suicide attempts	23 (32.9%)	10 (20.4%)	*p*= .15, OR= 1.90, 95% CI [0.76, 5.04]
*Lifetime comorbidities [n (%)]*			
Post-traumatic stress disorder	23 (32.8%)	5 (10.2%)	***p*= .004, OR= 4.26, 95% CI [1.42, 15.61]** **
Other anxiety disorders^1^	27 (38.6%)	11 (22.4%)	*p*= .07, OR= 2.15, 95% CI [0.89, 5.50] ^#^
Eating disorders^2^	13 (18.6%)	1 (2.0%)	N/A
Attention deficit hyperactivity disorder	13 (18.6%)	17 (34.7%)	*p*= .055, OR= 0.43, 95% CI [0.17, 1.08] ^#^
Alcohol abuse	11 (15.7%)	8 (16.3%)	*p*> .99, OR= 0.95, 95% CI [0.32, 2.99]
Alcohol dependence	8 (11.4%)	11 (22.4%)	*p*= .13, OR= 0.45, 95% CI [0.14, 1.35]
Substance abuse^3^	11 (15.7%)	10 (20.4%)	*p*= .62, OR= 0.73, 95% CI [0.25, 2.12]
Cannabis abuse	8 (11.4%)	6 (12.2%)	*p*> .99, OR= 0.92, 95% CI [0.26, 3.48]
Substance dependence^4^	12 (17.1%)	14 (28.6%)	*p*= .18, OR= 0.52, 95% CI [0.19, 1.37]
Cannabis dependence	5 (7.1%)	9 (18.4%)	*p*= .08, OR= 0.34, 95% CI [0.08, 1.24] #

Genders were compared with Wilcoxon test for continuous variables or Fisher’s exact test for nominal variables.

Abbreviations: CI= confidence intervals; HDRS= total score of 29-item Hamilton depression rating scale; N/*A*= non-applicable due to low n of participants in both genders; OR= odds ratio; SD= standard deviation; YMRS= total score of Young mania rating scale.

1 Other anxiety disorders include: generalized anxiety disorder, obsessive-compulsive disorder, social phobia, specific phobia, panic disorder

2 Eating disorders include: binge eating disorder, anorexia nervosa, bulimia nervosa and eating disorders not otherwise specified

3 Substance abuse include abuse for: cannabis (*n* = 14, 66.7%), cocaine (*n* = 4, 19.0%), hallucinogens (*n* = 2, 9.5%), opioids (*n* = 2, 9.5%), polysubstance (*n* = 1, 4.8%) and stimulants (*n* = 1, 4.8%).

4 Substance dependence include dependence for: cannabis (*n* = 14, 53.8%), cocaine (*n* = 12, 46.1%), hallucinogens (*n* = 2, 7.7%), opioids (*n* = 6, 23.1%), polysubstance (*n* = 8, 30.8%), sedatives (*n* = 2, 7.7%) and stimulants (*n* = 3, 11.5%).

** *p*< 0.01.

* *p*< 0.05.

# 0.1 < *p* > 0.05.

**Table 2:** Results of the association between the total score of the Childhood Trauma Questionnaire and clinical features in BD participants.

Clinical features	Number of participants	Total model	Main effect of clinical features
Mood state compared to Euthymic Depressed / Manic & hypomanic / Mixed	Euthymic= 55, Depressed= 33 Manic & hypomanic= 18 Mixed = 15	F_4,114_= 11.22, *p*< .001	***B* = 10.63** / 10.45 / **29.92, SE= 4.25** / 5.19 / **5.61, 95% CI [2.21, 19.05** / 0.17, 20.72 / **18.81, 41.03], p_uncorr_= 0.014** / 0.046 / **<0.001, p_FDR_ = 0.034** * / 0.084 # / **<0.001** ***
HDRS	*n* = 119	F_2,116_= 11.35, *p*< .001	***B* = 0.58, SE= 0.19, 95% CI [0.20, 0.96], p_uncorr_= 0.003, p_FDR_= 0.010** *
YMRS	*n* = 119	F_2,116_= 11.89, *p*< .001	***B* = 1.05, SE= 0.33, 95% CI [0.40, 1.70], p_uncorr_= 0.002, p_FDR_= 0.010** *
Age of first mood symptoms	*n* = 119	F_2,116_= 9.32, *p*< .001	***B*= −0.52, SE= 0.22, 95% CI [−0.95, −0.08], p_uncorr_= 0.019, p_FDR_= 0.043** *
# hospitalizations	*n* = 119	F_2,116_= 11.46, *p*< .001	***B* = 1.90, SE= 0.62, 95% CI [0.68, 3.12], p_uncorr_= 0.002, p_FDR_= 0.010** *
# depressive episodes	*n* = 119	F_2,116_= 9.98, *p*< .001	***B* = 0.61, SE= 0.23, 95% CI [0.15, 1.07], p_uncorr_= 0.01, p_FDR_= 0.029** *
# manic episodes	*n* = 119	F_2,116_= 8.59, *p*< .001	*B* = 0.45, SE= 0.22, 95% CI [0.02, 0.88], p_uncorr_= 0.04, p_FDR_= 0.080 #
Rapid cycling	*n* = 119	F_2,116_= 6.30, *p*= .002	*B* = 1.63, SE= 4.07, 95% CI [−6.44, 9.69], p_uncorr_= 0.69, p_FDR_= 0.73
Lifetime psychosis	*n* = 119	F_2,116_= 6.41, *p*= .002	*B*= −2.44, SE= 4.06, 95% CI [−10.49, 5.60], p_uncorr_= 0.55, p_FDR_= 0.61
Suicide attempts	*n* = 119	F_2,116_= 11.29, *p*< .001	***B* = 12.71, SE= 4.19, 95% CI [4.40, 21.01], p_uncorr_= 0.003, p_FDR_= 0.010** *
Post-traumatic stress disorder ^A^	Yes= 23 No= 47	F_1,68_= 23.5, *p*< .001	***B* = 25.68, SE= 5.30, 95% CI [15.11, 36.25], p_uncorr_< 0.001, p_FDR_ < 0.001** ***
Other anxiety disorders^1^	Yes= 38 No= 81	F_2,116_= 6.23, *p*= .003	*B* = 0.65, SE= 4.20, 95% CI [−7.67, 8.98], p_uncorr_= 0.87, p_FDR_> 0.99
Eating disorders^2, A^	Yes= 13 No= 57	F_1,68_= 1.97, *p*= .16	*B*= −10.28, SE= 7.32, 95% CI [−24.88, 4.32], p_uncorr_= 0.16, p_FDR_= 0.23
Attention deficit hyperactivity disorder	Yes= 30 No= 89	F_2,116_= 7.22, *p*= .001	*B*= −6.06, SE= 4.49, 95% CI [−14.96, 2.83], p_uncorr_= 0.18, p_FDR_= 0.24
Alcohol abuse^A^	Yes= 11 No= 59	F_1,68_= 1.28, *p*= .26	*B*= −8.88, SE= 7.86, 95% CI [−24.56, 6.80], p_uncorr_= 0.26, p_FDR_= 0.33
Alcohol dependence^B^	Yes= 11 No= 38	F_1,47_= 0.91, *p*= .35	*B* = 5.15, SE= 5.39, 95% CI [−5.70,15.99], p_uncorr_= 0.34, p_FDR_= 0.40
Substance abuse^3^	Yes= 21 No= 98	F_2,116_= 8.18, *p*< .001	*B* = 9.43, SE= 5.00, 95% CI [−0.48, 19.34], p_uncorr_= 0.062, p_FDR_= 0.095 #
Substance dependence^4^	Yes= 26 No= 93	F_2,116_= 8.21, *p*< .001	*B* = 8.83, SE= 4.65, 95% CI [−0.38, 18.03], p_uncorr_= 0.06, p_FDR_= 0.095 ^#^

All results present main effect of the clinical features controlled for gender.

Abbreviations: HDRS= total score of 29-item Hamilton depression rating scale; p_FDR_ = alpha with a false discovery rate (FDR) correction for multiple comparisons; p_uncorr_= alpha without correction for multiple comparisons; SE= standard error; YMRS= total score of Young mania rating scale.

A Analyses done only in women.

B Analyses done only in men.

1 Other anxiety disorders include: generalized anxiety disorder, obsessive-compulsive disorder, social phobia, specific phobia, panic disorder.

2 Eating disorders include: binge eating disorder, anorexia nervosa, bulimia nervosa and eating disorders not otherwise specified.

3 Substance abuse include abuse for: cannabis, cocaine, hallucinogens, opioids, polysubstance and stimulants.

4 Substance dependence include dependence for: cannabis, cocaine, hallucinogens, opioids, polysubstance, sedatives and stimulants.

*** *p*< 0.001.

* *p*< 0.05.

# 0.1 < *p* > 0.05.
