# Supplementary material for: Gender-related association among childhood maltreatment, brain structure and clinical features in bipolar disorder
Source: Eur Neuropsychopharmacol. Author manuscript; Available in PMC 2022 Oct 25. (PMC9593266; doi:10.1016/j.euroneuro.2022.07.186)
Supplement: SI — Supplementary Table 1 Medication of the Participants with Bipolar Disorder. Supplementary Table 2 Total score and subscale scores of Childhood Trauma Questionnaire; descriptive and statistical comparison for diagnosis and gender. Supplementary Table 3 Significant results of the association between the clinical features and brain regions by gender. [file NIHMS1841666-supplement-SI.docx]

**Supplementary Results**

After exclusion of the 9 subjects with more recent alcohol/substance abuse/dependence (women with BD, n=68, men with BD, n=42), the findings of associations between total score of the Childhood Trauma Questionnaire (CTQ-tot) and brain regions by gender were similar (left hippocampus, total model: F_5,104_=16.42, p<.001, interaction with gender: B=-9.14, SE=3.67, 95% CI[-16.42,-1.85], p=.014; left frontal pole: total model: F_5,104_=12.24, p<.001, interaction with gender: B=0.48, SE=0.30, 95% CI[-0.12,1.08], p=.1; right superior frontal: total model: F_5,104_=26.29, p<.001, interaction with gender: B=16.77, SE=8.05, 95% CI[0.80,32.73], p=.040).

Exploratory analyses of CTQ subscales and brain regions in BD participants showed significant associations as follows: for women left hippocampus with sexual abuse (total model: F_3,66_=13.6, p<.001, main effect: B=-16.58, SE=5.65, 95% CI[-27.86,-5.29], p=.005); for men left frontal pole with emotional neglect (total model: F_3,45_=14.16, p<.001, main effect: B=-2.15, SE=0.66, 95% CI[-3.48,-0.82], p=.002), physical neglect (total model: F_3,45_=10.82, p<.001, main effect: B=-2.74, SE=1.32, 95% CI[-5.41,-0.07], p=.044) and emotional abuse (F_3,45_=13.52, p<.001, main effect: B=-2.05, SE=0.67, 95% CI[-3.39,-0.70], p=.004), and right superior frontal surface area with the emotional neglect (total model: F_3,45_=20.57, p<.001, main effect: B=-49.97, SE=19.97, 95% CI[-90.19,-9.75], p=.016), physical abuse (total model: F_3,45_=19.01, p<.001, main effect: B=-54.69, SE=27.29, 95% CI[-109.66,0.28], p=.05) and sexual abuse (total model: F_3,45_=21.25, p<.001, main effect: B=-69.98, SE=26.01, 95% CI[-122.37,-17.59,], p=.01).

**Supplementary Table 1** Medication of the Participants with Bipolar Disorder.

|  | **Women (n= 70)** | **Men (n= 49)** | **Statistical comparison** |
| --- | --- | --- | --- |
| *Medication [n (%)]* ^1^ | | | |
| Unmedicated | 24 (34.3%) | 20 (40.8%) | p= .56, OR= 1.32, 95% CI [0.58, 3.00] |
| Levothyroxine | 2 (2.9%) | 2 (4.1%) | N/A |
| Thiamazole | 1 (1.4%) | 0 (0%) | N/A |
| *Indication-based* |  |  |  |
| Antipsychotics ^2^ | 19 (27.1%) | 20 (40.8%) | p= .16, OR= 0.54, 95% CI [0.23, 1.26] |
| Anticonvulsants | 24 (34.3%) | 14 (28.6%) | p= .55, OR= 1.30, 95% CI [0.55, 3.14] |
| Antidepressants ^3^ | 22 (31.4%) | 6 (12.2%) | **p= .017, OR= 3.25, 95% CI [1.14, 10.75] *** |
| Lithium | 10 (14.3%) | 7 (14.3%) | p> .99, OR= 1.00, 95% CI [0.31, 3.36] |
| Benzodiazepines | 17 (24.3%) | 6 (12.2%) | p= .16, OR= 2.28, 95% CI [0.77, 7.71] |
| Stimulants | 8 (11.4%) | 3 (6.1%) | p= .52, OR= 1.97, 95% CI [0.44, 12.14] |
| *Pharmacology-based* |  |  |  |
| Norepinephrine | 0 (0%) | 1 (2.0%) | N/A |
| Serotonin | 14 (20%) | 4 (8.2%) | p= .12, OR= 2.79, 95% CI [0.80, 12.46] |
| Dopamine | 2 (2.8%) | 1 (2.0%) | N/A |
| Norepinephrine, Serotonin | 4 (5.7%) | 2 (4.1%) | N/A |
| Norepinephrine, Dopamine | 13 (18.6%) | 3 (6.1%) | p= .059, OR= 3.46, 95% CI [0.88, 20.08] **^#^** |
| Serotonin, Dopamine | 9 (12.8%) | 8 (16.3%) | p= .60, OR= 0.76, 95% CI [0.24, 2.46] |
| Norepinephrine, Serotonin, Dopamine | 1 (1.4%) | 3 (6.1%) | N/A |
| Dopamine, Serotonin, Noradrenaline | 10 (14.3%) | 9 (18.4%) | p= .61, OR= 0.74, 95% CI [0.25, 2.27] |
| GABA | 19 (27.1%) | 6 (12.2%) | p= .067, OR= 2.65, 95% CI [0.91, 8.85] **^#^** |
| Glutamate | 22 (31.4%) | 14 (28.6%) | p= .84, OR= 1.14, 95% CI [0.48, 2.78] |
| GABA, Glutamate | 3 (4.3%) | 0 (0%) | N/A |
| Histamine | 2 (2.8%) | 3 (6.1%) | N/A |
| Lithium | 10 (14.3%) | 7 (14.3%) | p> .99, OR= 1.00, 95% CI [0.31, 3.36] |
| Other ^4^ | 4 (5.7%) | 3 (6.1%) | N/A |

^1^ Psychotropic medication were classified based on both “indication” and “pharmacology” following Neuroscience-based Nomenclature terminology; ^2^ Antipsychotics include: 1^st^ and 2^nd^ generation antipsychotics; ^3^ Antidepressants include: selective serotonin reuptake inhibitor, serotonin-norepinephrine reuptake inhibitor, and other antidepressants including bupropion, mirtazapine and trazodone. ^4^ Other include: opioid and acetylcholine pharmacology.

Genders were compared with Fisher’s exact test for nominal variables. * p< .05, **^#^** .1 < p > .05

Abbreviations: CI= confidence interval; GABA= gamma-Aminobutyric acid; N/A= non-applicable due to low n of subjects in both genders; OR= odds ratio.

**Supplementary Table 2** Total score and subscale scores of the Childhood Trauma Questionnaire; descriptive and statistical comparison for diagnosis and gender.

|  | **BD**  **women**  **(n= 70)** | **BD**  **women**  **(n= 49)** | **HC women**  **(n= 59)** | **HC**  **men**  **(n= 58)** | **Statistical comparison^A^** |
| --- | --- | --- | --- | --- | --- |
| CTQ-tot | 55.8±24.0 | 41.9±15.7 | 33.1±8.8 | 33.6±9.1 | B=-14.34, SE=4.25, 95% CI [-22.71,-5.97], p<.001*** |
| *range* | 25-116 | 25-85 | 25-64 | 25-67 |  |
| Emotional Abuse | 14.2±6.5 | 9.9±5.1 | 7.1±2.8 | 7.1±2.7 | B=-4.31, SE=1.23, 95% CI [-6.72,-1.89], p<.001*** |
| *range* | 5-25 | 5-25 | 5-18 | 5-16 |  |
| Physical Abuse | 9.5±6.0 | 7.3±3.9 | 5.8±1.7 | 6.3±2.4 | B=-2.75, SE=1.04, 95% CI [-4.81,-0.69], p=.011* |
| *range* | 5-25 | 5-25 | 5-13 | 5-21 |  |
| Sexual Abuse | 9.5±6.8 | 6.6±4.1 | 5.9±3.1 | 5.1±0.5 | B=-2.14, SE=1.17, 95% CI [-4.44,0.17], p=.07^#^ |
| *range* | 5-25 | 5-25 | 5-22 | 5-8 |  |
| Emotional Neglect | 13.6±6.1 | 10.4±5.3 | 8.1±3.3 | 8.7±4.2 | B=-3.63, SE=1.28, 95% CI [-6.16,-1.11], p=.004** |
| *range* | 5-25 | 5-23 | 5-16 | 5-25 |  |
| Physical Neglect | 9.0±4.4 | 7.8±2.7 | 6.0±1.8 | 6.3±2.1 | B=-1.51, SE=0.80, 95% CI [-3.09,0.07], p=.06^#^ |
| *range* | 5-25 | 5-14 | 5-12 | 5-15 |  |

^A^Report of the interaction effect between gender and diagnosis

All values are mean±standard deviation; ******* p< .001 ****** p< .01; ***** p< .05; **^#^** .1 < p > .05.

Abbreviations: CI= confidence interval; CTQ-tot= total score of Childhood Trauma Questionnaire; SE= Standard Error.

**Supplementary Table 3** Significant results of the association between the clinical features and brain regions by gender.

| **Clinical Feature** | **Number of participants** | **Total model** | **Main effect of clinical features** |
| --- | --- | --- | --- |
| *left hippocampus volume for women with BD* | | | |
| Mood state compared to Euthymic  Depressed /  Manic & hypomanic /  Mixed | Euthymic= 26  Depressed= 22  Manic & hypomanic= 11  Mixed= 11 | F_6,63_= 7.09, p< .001 | B= -54.28 / -211.11 / **-306.30**, SE= 94.93 / 122.60 / **130.90**, 95% CI [-243.98,135.41 / -456.08, 33.79 / **-567.91, -44.77**], p= 0.6 / 0.09 / **0.02** ***** |
| YMRS | n= 70 | F_4,65_= 9.97, p< .001 | **B= -13.91, SE= 6.68, 95% CI [-27.26, -0.56], p= .04 *** |
| Age of first mood symptoms | n= 70 | F_4,65_= 9.47, p< .001 | B= -10.61, SE= 6.14, 95% CI [-22.87, 1.64], p= .09 ^#^ |
| *left frontal pole surface area for men with BD* | | | |
| YMRS | n= 49 | F_4,44_= 12.56, p< .001 | **B= -1.42, SE= 0.71, 95% CI [-2.85, 5.89^10^-4^], p= .05 *** |
| *right superior frontal surface area for men with BD* | | | |
| Age of first mood symptoms |  | F_4,44_= 19.95, p< .001 | **B= 33.97, SE= 13.08, 95% CI [7.61, 60.33], p= .013 *** |
| Number of manic episodes | n= 49 | F_4,44_= 19.95, p< .001 | B= -21.32, SE= 12.28, 95% CI [-46.07, 3.43], p= .09 ^#^ |

Main effect of clinical features on the brain structures (controlling for CTQ-tot) is presented since all interactions with CTQ-tot were not significant (p’s>.1). ****** p< .01; ***** p< .05, **^#^** .1 < p > .05, uncorrected for multiple comparisons.

Abbreviations: CI= confidence interval; SE= standard error; YMRS= Young mania rating scale.
